# Optical Co-registration of MRI and On-scalp MEG

**DOI:** 10.1038/s41598-019-41763-4

**Published:** 2019-04-02

**Authors:** Rasmus Zetter, Joonas Iivanainen, Lauri Parkkonen

**Affiliations:** 1 Department of Neuroscience and Biomedical Engineering, Aalto University School of Science, FI-00076 Aalto, Finland; 2 Aalto NeuroImaging, Aalto University, FI-00076 Aalto, Finland

## Abstract

To estimate the neural generators of magnetoencephalographic (MEG) signals, MEG data have to be co-registered with an anatomical image, typically an MR image. Optically-pumped magnetometers (OPMs) enable the construction of on-scalp MEG systems providing higher sensitivity and spatial resolution than conventional SQUID-based MEG systems. We present a co-registration method that can be applied to on-scalp MEG systems, regardless of the number of sensors. We apply a structured-light scanner to create a surface mesh of the subject’s head and the sensor array, which we fit to the MR image. We quantified the reproducibility of the mesh and localised current dipoles with a phantom. Additionally, we measured somatosensory evoked fields (SEFs) to median nerve stimulation and compared the dipole positions between on-scalp and SQUID-based systems. The scanner reproduced the head surface with <1 mm error. Phantom dipoles were localised with 2.1 mm mean error. SEF dipoles corresponding to the P35m response for OPMs were well localised to the somatosensory cortex, while SQUID dipoles for two subjects were erroneously localised to the motor cortex. The developed co-registration method is inexpensive, fast and can easily be applied to on-scalp MEG. It is more convenient than traditional co-registration methods while also being more accurate.

## Introduction

Magnetoencephalography (MEG) is a non-invasive functional neuroimaging method for investigating electric neuronal activity of the the living human brain^1^. MEG systems measure the magnetic field produced by neural currents in the brain using sensors positioned around the head.

So far, the sensor type employed for MEG has almost exclusively been the low-T_c_ superconducting quantum interference device (SQUID). Low-T_c_ SQUIDs require a cryogenic temperature that is typically attained by immersing SQUIDs in liquid helium (T ≈ 4.2 K ≈ −269 °C). The necessary thermal insulation keeps SQUIDs at least 2 cm away from the scalp in most commercial systems and makes the construction of adaptable sensor arrays extremely challenging. Since sensitivity and spatial resolution are related to the distance between the sources and the sensors, the need of cryogenics eventually results in a considerable loss of signal amplitude and spatial resolution^2,3^.

New sensor technologies with sensitivity high enough for MEG have emerged recently; optically-pumped magnetometers (OPMs)^4,5^ and high-T_c_ SQUIDs^6^ hold promise as alternatives to low-T_c_ SQUIDs. These new sensor types do not require the same degree of thermal insulation as low-T_c_ SQUIDs and can thus be placed almost directly on the scalp, considerably boosting both the sensitivity to neural sources as well as spatial resolution.

In order to determine the origins of neuromagnetic signals measured with MEG, one needs to incorporate anatomical information, typically obtained from structural MRI. To be able to combine data from the two imaging modalities, their coordinate systems need to be aligned, i.e., co-registered. Accurate co-registration is particularly important in on-scalp MEG^7^ due to its high spatial resolution^2,3^.

The current standard co-registration method in SQUID-based MEG relies on the combination of head position indicator (HPI) coils attached to the participant’s head and a pen-like electromagnetic 3D digitiser. Prior to MEG measurements, the positions of the HPI coils as well as a set of anatomical landmarks on the head are digitised. To localise the HPI coils with respect to the MEG sensors, known currents are driven into the coils either sequentially or at different frequencies prior to or continuously during MEG measurements and a magnetic dipole model representing each coil is fitted to the acquired MEG sensor signals. Finally, the actual co-registration is performed by aligning the HPI-coil locations as determined by the MEG system with those determined by digitisation, and aligning the digitised anatomical landmarks with the same landmarks in the MR image.

The accuracy of the co-registration can be improved by digitising not only the landmarks but a larger set of points on the head surface. Due to the need to manually digitise each point, their number is limited. For the same reason, the number of HPI coils is typically no more than 5. However, with optical scanning methods, one can obtain several orders of magnitude more points in less time than used with current methods. Additionally, the accurate localisation of the HPI coils requires a MEG sensor array with extensive coverage and a large number of channels. Thus, using HPI coils with the current, early-stage on-scalp MEG systems with only a few channels is not feasible.

Here, we describe a co-registration method that employs a commercial, consumer-grade structured-light scanner that is suitable for an on-scalp MEG system with a partially rigid sensor array. We validate the co-registration method both in terms of reproducibility and accuracy, using phantom measurements as well as a human experiment.

## Materials and Methods

### Structured-light scanner

We applied a consumer-grade structured-light scanner (Occipital Inc., San Francisco, CA, USA) to digitise the head surface of the subject as well as the MEG sensor helmet. The structured-light scanner functions by projecting a pattern of infrared light onto an object, which is then detected by a camera at a known distance from the projector. The three-dimensional shape of the scanned object can then be determined based on the apparent distortion of the pattern as seen by the camera. The scanner captures both colour and depth data at a frame rate of 30 Hz, with each frame being co-registered to the previous one in real time. The scanner is connected to a tablet computer (iPad; Apple Inc., Cupertino, CA, USA), which can both function as an operator display and compute the digitised surface mesh in real time. At a typical working distance of 50 cm, the scanner has a vendor-specified point accuracy of 0.8 mm^8^.

### On-scalp MEG system

We applied the structured-light scanner to an on-scalp MEG system comprising nine QuSpin ZF-OPM sensors (QuSpin Inc., Louisville, CO, USA) in a partially rigid array, where each sensor measures the magnetic field component approximately normal to the head surface. For a detailed description of the system, including data acquisition electronics, see the work by Iivanainen and colleagues^9^. The sensors are mounted in a 3D-printed helmet with geometry identical to that of the Elekta Neuromag Vectorview and TRIUX (MEGIN/Elekta Oy, Helsinki, Finland) 306-channel SQUID-based MEG systems. Individual OPMs are placed into sockets, whose positions and orientations correspond to those of the above SQUID systems, and inserted until touching the head of the subject. The insertion depth is manually measured for each sensor.

**Figure 1:**
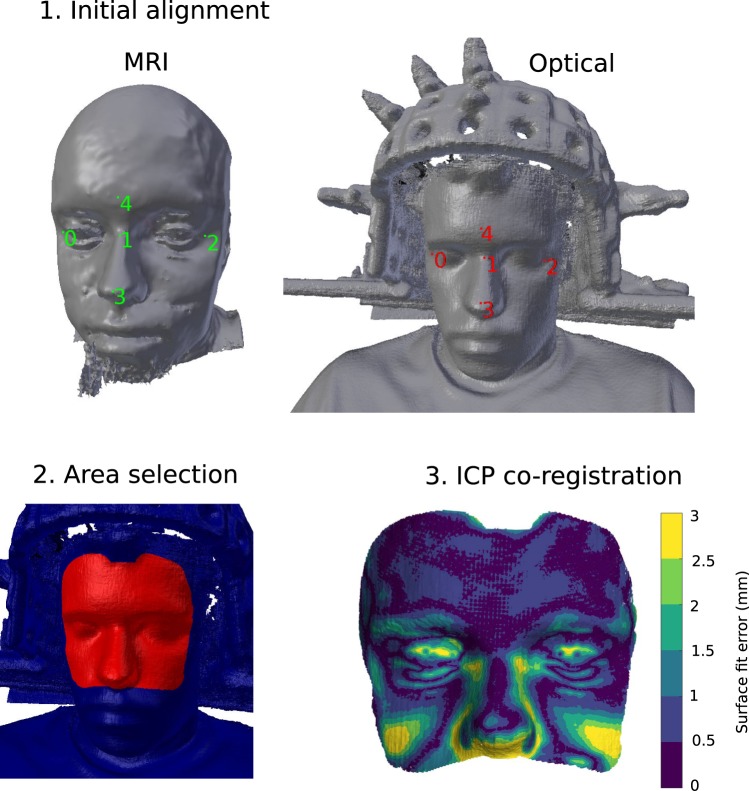
Optical co-registration procedure: 1. Initial mesh alignment with manually selected fiducial points, shown as coloured numerals, on the MR (left, green) and structured-light scan (right, red) meshes. Dummy sensors used to fix the head position are seen on either side of the head in the optical scan. 2. Selection of the co-registration area, selected area shown in red. 3. Automatic ICP-based co-registration and visualisation of the surface fit error.

For the human measurements, the helmet was attached to the subject chair, and two dummy sensors were used to fix the subject’s head to the sensor helmet in order to minimise movement (Fig. 1, top panel).

### SQUID-based MEG system

For validation and comparison purposes, MEG was also recorded using a 306-channel SQUID-based Elekta Neuromag VectorView MEG system. Data were recorded using a sampling frequency of 1 kHz. For the analyses in this work, both SQUID magnetometers and gradiometers were used.

### MEG-MRI co-registration process

After positioning the subject in the OPM-MEG system, an optical scan is performed by moving the structured-light scanner around the subject at a distance of approximately 50 cm. The digitised surface is visualised in real time, enabling the operator to perform quick corrections and fill in any gaps in scan coverage by going over specific areas again. The scan takes approximately 30 s to perform, depending on the desired coverage. While the real-time constructed mesh functions as a good reference, it has limited resolution. For increased accuracy and resolution, we apply an offline mesh reconstruction algorithm found in the Skanect software package (Occipital Inc., San Francisco, CA, USA) to compute the final surface mesh (Object mode, ‘High’ resolution setting).

Using the *mkheadsurf* tool (with default parameters) included in the FreeSurfer software package^10–12^, a corresponding scalp surface mesh is extracted from the structural MR image.

The co-registration algorithm was implemented as a plug-in to Blender (https://blender.org), an open-source 3D creation software suite. The co-registration process is initialised by a rough alignment of the optical scan mesh and the MR scalp mesh by manually selecting and aligning a small number of fiducial points on both meshes (Fig. 1, top panel).

Since the optical scan includes both the subject’s head and the MEG helmet with sensors, the area (vertices of the mesh) used for co-registration with the MR image must be restricted. This is done manually by “painting” over the desired parts of the mesh with the computer mouse (Fig. 1, bottom-left panel). Any parts of the optical scan can be explicitly excluded in the same manner. Being able to quickly constrain the areas used for co-registration makes it easy to exclude areas with visible artifacts. For co-registration of the MR scalp mesh and optical scan mesh, only the upper face and forehead areas are used. Previous work has shown that using face data for high-density meshes is sufficient for accurate co-registration of EEG and MEG^13–16^.

After the initial alignment and vertex selection, the iterative closest point (ICP) algorithm with point-to-point minimisation is run for up to 100 iterations to automatically co-register the meshes. If the mesh translation is less than 0.1 mm in an iteration, the algorithm is stopped (typically within 10 iterations; see Fig. 1 for a visualisation of the resulting co-registration fit).

After co-registering the optical scan and the MR image, the same procedure is repeated to align the known geometry of the MEG helmet to the optical scan (now in MR coordinates). Manually-selected points along the front edge the sensor helmet are used for the initial alignment. The entire outer surface visible in the scan, excluding areas with sensors, is employed in the ICP alignment procedure. The coordinates and orientations of the sensor slots of the helmet are then retrieved from the helmet model, and the manually-measured insertion depths are taken into account to get the actual sensor positions.

## Experiments

### Phantom experiment: Reproducibility

To attain a baseline scanner performance estimate, we scanned a head-shaped polystyrene phantom five times, with each scan taking approximately 45 s. These five repetitions were thereafter co-registered using the process described above. After co-registration, we used the initial scan as a baseline and for each vertex we computed the distance to the closest vertex in the subsequent scans.

We also repeated this experiment while only using the upper face and forehead areas of the phantom for co-registration to simulate the real-world use of the scanner. This scenario allows us to determine whether using only the facial area for co-registration may cause any co-registration error in other regions of the head.

### Phantom experiment: Dipole localisation using OPMs

To validate the co-registration methodology in a controlled manner, we applied a calibrated dry phantom (MEGIN/Elekta Oy, Helsinki, Finland) containing 32 sources at known positions within a head-sized hemisphere^17,18^. Each source is a current loop that comprises a 5-mm long tangential segment producing the field of a current dipole and two radial segments that generate a field corresponding to the volume-current field associated with that current dipole. Thus, the total field is that of a current dipole in spherical conducting medium.

Eight of these sources were sequentially energised with 2 cycles of a 20-Hz sinusoid with a peak amplitude of 500 nAm. This pulse was repeated 100 times while the produced magnetic field was measured using the OPM-MEG system. The sensors were positioned as to cover both negative and positive field maxima. This measurement procedure was repeated five times with slightly different positions of the phantom with respect to the sensors. For each repetition, the phantom and sensor helmet were co-registered using the structured-light scanner. The co-registration method was applied as it would be for human subjects, with a 3D model of the known geometry of the dry phantom replacing the MR scalp mesh.

### Human experiment: SEF measurements using OPMs and SQUIDs

We recorded somatosensory evoked fields (SEFs) from three subjects (all male, 26–33 years) using both the SQUID-based 306-channel system and our OPM-based MEG system^9^. For Subject 1, 9 OPMs were used while for Subjects 2 and 3, only 8 OPMs were available. The OPM measurement was performed twice for Subject 3; the sensor positions were adjusted based on the first measurement for better spatial coverage of the response, as proper spatial sampling is crucial with only eight sensors. Somatosensory responses were produced by transcutaneous electric median nerve stimulation delivered to the left median nerve at the wrist using 7.5-mA 200-*μ*s current pulses. 200 trials were recorded with an inter-trial interval of 2.5 s with ±0.2 s uniformly distributed jitter. The experimental design took into consideration the code of ethics as defined in the World Medical Association’s Declaration of Helsinki, and the study was approved by the Aalto University Ethics Committee. Informed consent was obtained from the participants.

For the SQUID measurements, co-registration was performed prior to MEG recording using five HPI coils and a Polhemus Isotrak electromagnetic digitiser: the nasion and preauricular points were used as initial fiducial points for alignment with the MR image, as well as 100–200 scalp points covering the scalp, forehead and bridge of the nose. For the OPM-based system, the structured-light scanning co-registration presented in this work was applied. The OPMs were placed above the somatomotor areas of the hemisphere contralateral to the stimulation, such that they would measure both the positive and negative field maxima while also providing good spatial resolution.

T1-weighted structural MR images were used in source modeling and co-registration. MR images were acquired using a 3 T MAGNETOM Skyra whole-body scanner (Siemens Healthcare, Erlangen, Germany) and a 32-channel receiving head coil. The subjects wore earplugs and tightly packed foam covers over their ears for additional hearing protection and to minimise head movement. The T1-weighted 3D-MPRAGE structural image was acquired using the following parameters: TR 2530 ms, TE 3.3 ms, TI 1100 ms, FA of 7°, in-plane FOV 256 × 256 mm, 1 mm isotropic voxels, 176 contiguous slices and a GRAPPA factor of 2.

The FreeSurfer software package^10,12,19^ was used for pre-processing the MRI and for segmentation of the cortical surfaces. The skull and scalp surfaces were segmented using the watershed approach^20^ implemented in FreeSurfer and MNE software^21^. These surfaces were thereafter decimated to obtain three boundary element meshes (2562 vertices per mesh).

MEG data were bandpass-filtered to 0.1–100 Hz with notches at 50 and 100 Hz in both measurements, epochs were manually inspected for artifacts, and thereafter averaged. For the SQUID measurements, external magnetic interference was suppressed using the temporally-extended signal-space separation (tSSS) method^22^ implemented in the MaxFilter software (version 2.2; MEGIN/Elekta Oy, Helsinki, Finland).

A single equivalent current dipole was fitted to the P35m response peak. The noise covariance matrix used in the fitting process was computed using baseline data (500–50 ms preceding stimulation). All analyses were performed using the MNE-Python software^21^. Dipole fitting for the SQUID measurements was performed using all channels, including both magnetometers and gradiometers.

## Results

### Phantom experiment: Reproducibility

**Figure 2:**
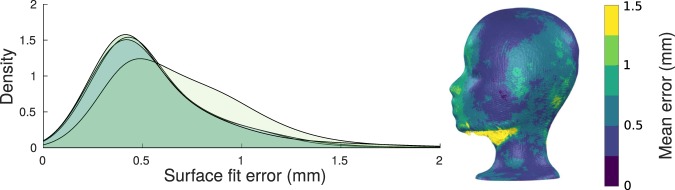
Reproducibility of the surface mesh reconstructed by the optical scanner. Distributions (left) and spatial locations (right) of errors across five scans of the same object. Each coloured density plot represents the error of one repetition.

The five optical scan meshes of the phantom head had vertex and triangle count ranges of 353,163–375,914 and 702,417–745,744, respectively. The mean surface fit error (i.e. reproducibility error) of the optical scanner was 0.87 mm across all repetitions and vertices (error distribution modes within 0.42–0.49 mm). As is evident from Fig. 2, the errors are larger in the area under the chin of the phantom head, which the scanner could image only at very oblique angles. This area with larger errors is also seen in the error distributions in Fig. 2 as the right-hand-side tail.

When only using the upper face and forehead area of the phantom for co-registration, the mean surface fit error over the entire head, across all repetitions, was 1.37 mm (error distribution modes within 0.43–0.48 mm). As seen in Fig. S1, the tails of the error distribution have grown due to the misalignment in the base (neck) of the phantom, while the head, including occipital areas, have mostly <1-mm error.

### Phantom experiment: Dipole localisation using OPMs

**Figure 3:**
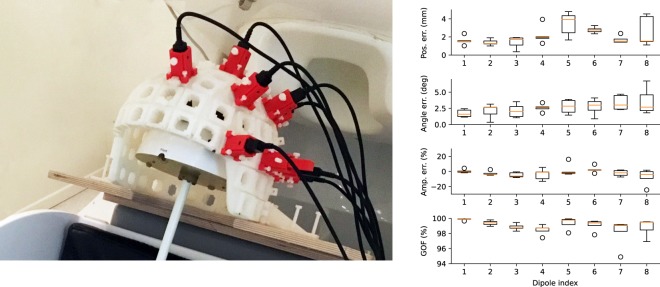
Left: Phantom measurement setup. Right: Phantom dipole localisation; absolute position, orientation and amplitude errors as well as goodness-of-fit (GOF).

The five optical scan meshes of the dry phantom and sensor helmet had vertex and triangle count ranges of 1,047,044–1,095,527 and 2,020,151–2,121,927, respectively. The accuracy of localising the dipolar current sources in the phantom is shown in Fig. 3 (right-hand panel). Across all dipoles, the absolute localisation error was 2.14 ± 1.07 mm (mean ± SD), the orientation error was 2.63° ± 1.22° (mean ± SD) and the amplitude error was −10.27 ± 21.17 nAm (the nominal amplitude was 500 nAm). Goodness-of-fit varied within the range 94.88–99.92% (98.98 ± 0.96%, mean ± SD).

### Human experiment: SEF measurements using OPMs and SQUIDs

**Figure 4:**
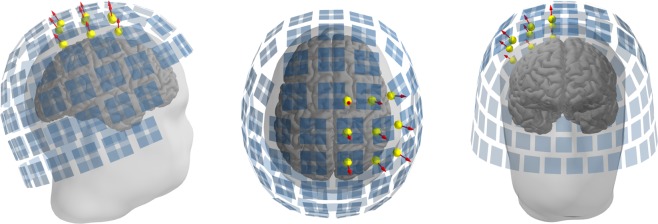
Positions of the OPM (yellow spheres; sensitive axes as red arrows) and SQUID (blue rectangles) sensors in the somatosensory measurement, Subject 1 shown as an example.

**Figure 5:**
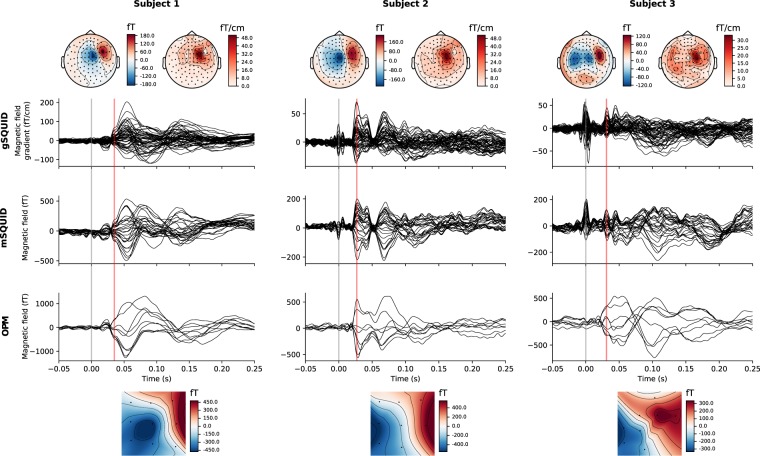
Somatosensory evoked fields for the OPM and SQUID measurements for all three subjects. Responses only at a subset of SQUID channels are shown for easier interpretation: 27 magnetometers (mSQUID) and 54 gradiometers (gSQUID) covering the right somatomotor cortex. The stimulus onset is indicated by a grey line. Equivalent current dipoles were fitted at the latency indicated by the red vertical line. Topographic maps at this latency for each sensor type are also shown (SQUID magnetometers and gradiometers above, OPMs below the SEF traces). For the SQUID gradiometer topographic maps, the RMS value of each gradiometer pair is shown. Stimulus artifacts can be seen in SQUID signals at 0 ms, especially for Subject 3.

**Figure 6:**
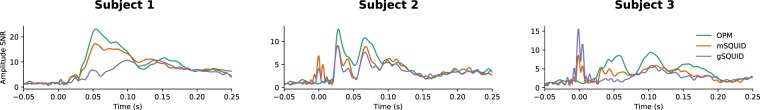
SNR estimates for OPM and SQUID SEF measurements. For the SQUID measurements, those *N* magnetometers (mSQUID) and gradiometers (gSQUID) with the highest baseline-normalised signal power were chosen interpretation, where *N* is the number of OPMs used for that subject. Stimulus artifacts can be seen in SQUID signals at 0 ms, especially for subject 3.

The optical scans used for co-registration had vertex and triangle count ranges of 803,480–1,006,181 and 1,568,590–1,940,677, respectively. The MRI scalp meshes had vertex and triangle count ranges of 265,239–325,196 and 535,784–671,250, respectively. The matching of the optical scan and MRI mesh of Subject 1 is illustrated in Fig. 1 (bottom-right panel). Co-registered OPM and SQUID sensor positions are shown in Fig. 4. The SEFs of both OPM and SQUID measurements for all subjects are seen in Fig. 5. Estimates of SEF SNR based on the global field power of the baseline-normalised evoked data are shown in Fig. 6 for both OPM and SQUID magnetometer data. For the SQUID measurements, the *N* magnetometers (mSQUID) and gradiometers (gSQUID) with the highest baseline-normalised signal power were chosen, where *N* is the number of OPMs used for that subject.

**Figure 7:**
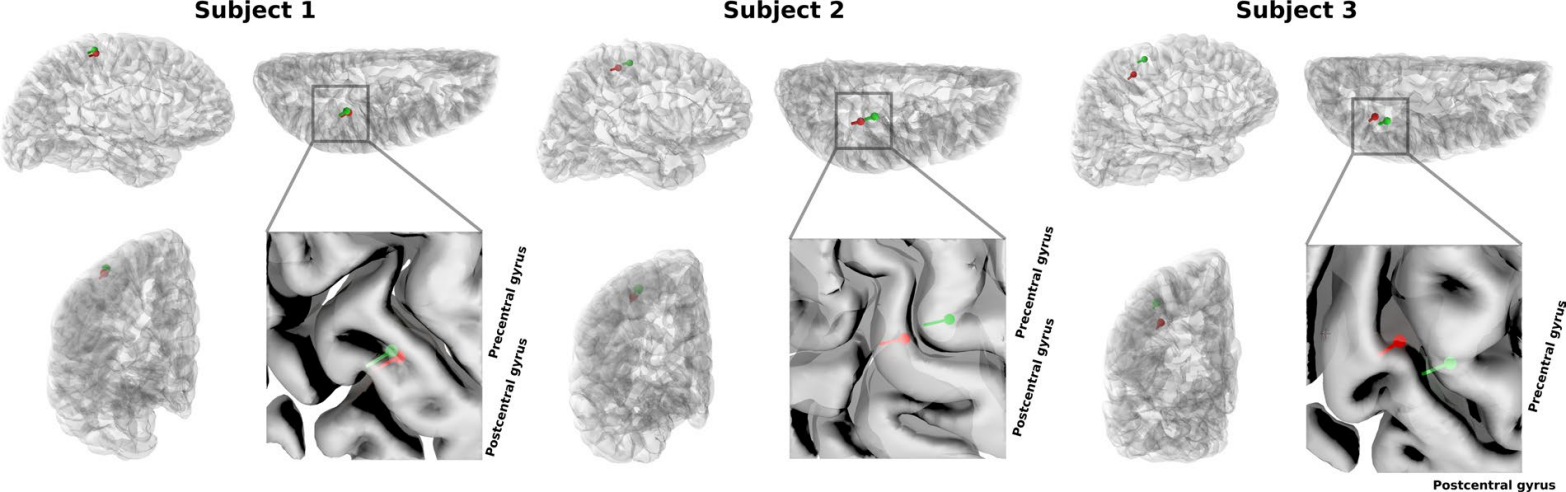
Locations of equivalent current dipoles representing the P35m somatosensory response peak visualised on orthogonal views (lateral, dorsal, rostral) of the right hemisphere. Both OPM (red) and SQUID (green) -based dipoles are shown. For more detailed visualisation, an enlarged section of the dorsal view is also shown.

A single equivalent current dipole was fitted to the P35m response peak for both OPM- and SQUID-MEG measurements. Characteristics of the fitted dipoles are shown in Table 1, and they are visualised in Fig. 7.

**Table 1:** SEF equivalent current dipole parameters for the P35m response peak.

Subject	Time (ms)	System	Amplitude (nAm)	GOF (%)	Position (x, y, z) (mm)	Δ Pos (mm)	Orientation (x, y, z)	Δ Ori (°)
1	35	OPM	15.1	95.3	41.2, −22.5, 80.7	2.9	0.44, −0.80, −0.40	2.6
		SQUID	11.1	78.3	40.3, −23.6, 83.2		0.48, −0.78, −0.41	
2	27	OPM	24.5	98.8	36.0, −41.1, 69.0	9.4	0.23, −0.93, −0.29	6.9
		SQUID	24.3	90.9	32.5, −33.0, 72.5		0.29, −0.94, −0.19	
3	31	OPM	17.4	96.4	36.8, −51.2, 37.7	15.0	0.45, −0.65, −0.61	18.9
		SQUID	11.0	62.7	40.2, −43.4, 50.0		0.34, −0.86, −0.38

## Discussion

Accurate co-registration of MEG and MRI is critical for reliable source estimation as shown in several studies concerning conventional SQUID-based MEG^15,23^, EEG^24,25^ and more recently also on-scalp MEG^7^. In this work, we present a co-registration method based on a structured-light scanner that can be applied to on-scalp MEG, but could also in principle be applied to SQUID-based MEG.

The traditional co-registration procedure based on HPI coils and an electromagnetic digitiser has been challenged during the past ten years by faster or more accurate digitisation and co-registration methods^14–16,24,26,27^. With the ongoing development and commoditization of consumer-grade 3D scanners, the use of optical co-registration has become increasingly attractive.

The accuracy of the optical scanner employed in this work seems to be satisfactory, with error levels at the sub-millimetre level (Fig. 2), which is on par to what is specified for the popular Polhemus electromagnetic digitiser^28^. However, the weakness of digitiser-based co-registration methods is their manual, operator-dependent nature; a human has to accurately move the digitiser to each reference point (without shifting the point itself). The digitisation of HPI coils is especially sensitive to operator error, as only five or even fewer HPI coils are typically used. In practice, the reported accuracy of Polhemus-based co-registration ranges from 3 to 7 mm^14,16,27,29^. We assume that these results would also extend to OPM-based MEG systems, as the errors in Polhemus-based co-registration are not dependent on the sensor type.

Optical co-registration methods also have limitations, the foremost of which is the line-of-sight issue. OPM sensors are typically closely spaced on the scalp, and together with their cables they present a complex surface that is not easy to digitise accurately, with complete coverage and without artifacts. Therefore, we register the rigid sensor helmet to MRI, and simply measure the insertion depth of each sensor into the helmet instead of digitising the location of each sensor directly.

Furthermore, when using optical co-registration, one needs to identify each sensor unambiguously. In our case, the use of a sensor helmet with discrete slots simplifies the matter; we log the assignment of sensors to the helmet slots for each measurement. In earlier EEG work, automated algorithms^13,30^, coloured markers^31^ or even timed LEDs^32^ have successfully been used to solve this problem.

Another co-registration method that turns the traditional use of HPI coils on its head was presented by Pfeiffer and colleagues^33^, who suggest using an array of small coils at known locations to localize the sensors. This approach is more suited for use with fully flexible sensor arrays than the approach we present in this work. However, as Pfeiffer and colleagues show, the locations and orientations of the reference coils must be known to a very high accuracy (preferably <1 mm, 1° error), and a link between their locations and the MR image must still be determined (e.g. using an optical scan or a digitiser). The method presented by Pfeiffer and colleagues could be used in conjunction with our optical scan method for quick, automated sensor depth measurement and helmet slot identification. This will be especially relevant as the number of sensors in the array grows.

Constraining the area in the MRI and optical surface scan used for co-registration is crucial to avoid artifacts. In addition, certain head surface features, such as the jaw, may be slightly different in the MRI and surface scan due to the difference in body orientation. Another evident difference is the compression of some facial features, especially in the cheeks, due to the padding used to minimise head movement during MRI acquisition. Facial areas such as the brow and forehead should function as more dependable areas for co-registration, as there the skin does not move as much in relation to the skull as in other areas.

Co-registration is not the only source of error in source estimation. In our experiment with the phantom, inaccurate sensor calibration and the small number of channels may have hampered dipole localisation. The phantom dipoles were localised, on average, with better than 2.5-mm accuracy, which is similar to what has been reported earlier when applying optical co-registration to whole-head SQUID-based MEG systems^14,16^ and significantly more accurate than these studies reported for electromagnetic digitiser-based co-registration.

Our somatosensory measurement revealed the typical sequence of evoked responses for such stimuli, as seen in Fig. 5. The first response appeared at approximately 20 ms after stimulus onset (N20m). As the N20m had surprisingly poor SNR in the SQUID measurements of Subjects 2 and 3, we concentrated on the stronger P35m response as it could be localized more accurately and thus probes co-registration accuracy better.

In spite of a somewhat biased SNR estimation procedure, in which the best SQUIDs from the whole-head array are compared to the OPMs, the OPMs had slightly higher SNR than SQUID magnetometers, which in turn had higher SNR than SQUID gradiometers (Fig. 6). This result shows the promise of on-scalp MEG, and agrees with the findings of simulation studies^2,3^ comparing conventional MEG and on-scalp MEG.

When using the entire SQUID array for localisation, OPM- and SQUID-localised P35m dipoles for Subject 1 match well, with less than 3-mm location difference. However, the SQUID-localised P35m dipoles for Subjects 2 and 3 are located within the precentral gyrus, i.e. the motor cortex, while the OPM-localised dipoles fall within the postcentral gyrus, i.e. the somatosensory cortex. Although the SQUID measurements were intended to serve as a reference for the OPM measurements, here we see that the OPM-localised dipoles may correspond to the known physiology better than the SQUID-localised dipoles.

For Subject 3, the OPM-localised dipole is quite deep, almost at the bottom of the sulcus. This is probably an error that may stem from the small number of sensors and the limited scalp coverage. As seen from the topographic maps in Fig. 5, Subject 3 shows some ipsilateral activity in the SQUID measurement which could affect the single-dipole fit. However, using only the channels on the contralateral side or alternatively employing a two-dipole fit did not alter the dipole location significantly in the anterior–posterior direction, even though it improved the goodness-of-fit.

It is possible that simultaneous activity within the motor cortex^34^ could bias the single-dipole fit in the anterior direction, but as this should be equally present in the OPM measurements at the same latency, it does not explain the discrepancy between OPM and SQUID measurements in Subjects 2 and 3. As our results in the SEF experiment show, the SQUID-localised responses are not necessarily the perfect ground truth: based on previous literature as well as the current results, the co-registration process used for the OPM system should be more accurate than that of the SQUID system. Previous work has shown that intersession variability of P35m localisation using SQUID-MEG can be ~5 mm^35^, and N20m localisation between different SQUID-based MEG systems can have a variability of ~8 mm^36^. As argued by Solomon and colleagues^35^, some of the variability in these results may stem from co-registration errors, as electromagnetic digitiser -based co-registration was used. Keeping these results in mind, the differences between OPM- and SQUID-derived dipole localisations in this work are in line with previous results. It would be of interest to perform a more focused, systematic experiment looking into the sources of localisation error for both OPMs and SQUIDs although that falls beyond the scope of the current study.

As the structured-light scanner acquires data at 30 Hz, it could be applied for continuous head tracking during measurements in the future. This could be done either using retroreflective markers attached to the head of the subject or using the entire digitised surface. Retroreflective markers would provide easily recognisable reference points that can be used to track head movements without any manual preprocessing. However, as the number of markers would be limited, they would have to be attached robustly to locations that do not move in relation to the head.

The methodology presented in this work can easily be applied to conventional SQUID-based MEG, and should have significant accuracy and speed benefits over the traditional digitiser-based approach. Similar methods applied to SQUID-based MEG have previously been presented by Bardouille and colleagues^14^, Hironaga and colleagues^15^ as well as Murthy and colleagues^16^.

In the future, as the number of sensors in OPM-based MEG system increases, optical surface scanning can also be used in conjunction with HPI coils for tried-and-true real-time head tracking. However, current-generation OPMs have a limited bandwidth only up to 150 Hz, meaning that only a small frequency window, which is close to that of physiological MEG data, is available for electromagnetic co-registration methods during MEG measurements.

## Conclusions

We present an optical co-registration method that can be applied to on-scalp MEG as well as to current conventional MEG systems. Based on comparison with previous reports, the optical co-registration method is more accurate and faster than conventional electromagnetic digitiser-based methods.

## Supplementary information

Supplementary information

## Data Availability

Data from the phantom experiment are available upon reasonable request. Data from the human experiment are not publicly available due to prohibition by Finnish law. As the consent given by the subject only applies to the specific study reported in our manuscript, no portion of the human data collected can be used or released for use by third parties.
