# Supplementary material for: Optical Co-registration of MRI and On-scalp MEG
Source: Sci Rep. 2019 Apr 2;9:5490. doi: 10.1038/s41598-019-41763-4 (PMC6445124; doi:10.1038/s41598-019-41763-4)
Supplement: Supplementary file 1 — Supplementary information [file 41598_2019_41763_MOESM1_ESM.pdf]

Optical Co-registration of MRI and On-scalp MEG Supplementary information

Rasmus Zetter[1,*], Joonas Iivanainen[1], and Lauri Parkkonen[1,2]

[1]Department of Neuroscience and Biomedical Engineering, Aalto University School of Science, FI-00076 Aalto, Finland
[2]Aalto NeuroImaging, Aalto University, FI-00076 Aalto, Finland
[*]rasmus.zetter@aalto.fi

ABSTRACT

To estimate the neural generators of magnetoencephalographic (MEG) signals, MEG data have to be co-registered with an anatomical image, typically an MR image. Optically-pumped magnetometers (OPMs) enable the construction of on-scalp MEG systems providing higher sensitivity and spatial resolution than conventional SQUID-based MEG systems. We present a co-registration method that can be applied to on-scalp MEG systems, regardless of the number of sensors. We apply a structured-light scanner to create a surface mesh of the subject's head and the sensor array, which we fit to the MR image. We quantified the reproducibility of the mesh and localised current dipoles with a phantom. Additionally, we measured somatosensory evoked fields (SEFs) to median nerve stimulation and compared the dipole positions between on-scalp and SQUID-based systems. The scanner reproduced the head surface with < 1 mm error. Phantom dipoles were localised with 2.1 mm mean error. SEF dipoles corresponding to the P35m response for OPMs were well localised to the somatosensory cortex, while SQUID dipoles for two subjects were erroneously localised to the motor cortex. The developed co-registration method is inexpensive, fast and can easily be applied to on-scalp MEG. It is more convenient than traditional co-registration methods while also being more accurate.

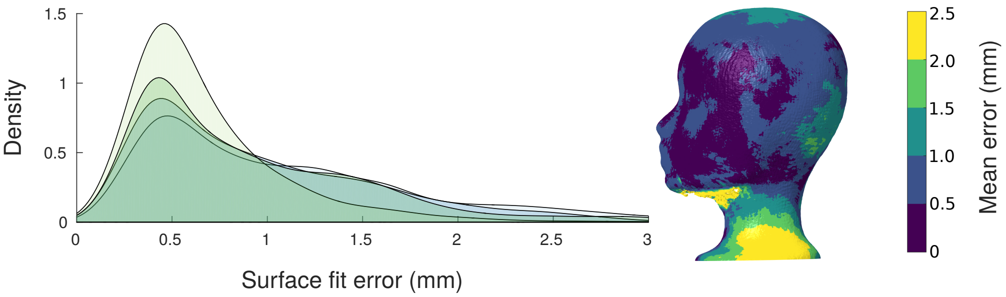

Figure S1. Reproducibility of the surface mesh reconstructed by the optical scanner when only the upper part of the face and forehead are used for co-registration. Distributions (left) and spatial locations (right) of errors across five scans of the same object. Each coloured density plot represents the error of one repetition.
